# Secondary prevention in coronary artery disease: development and content validity of educational messages for mobile phones

**DOI:** 10.1590/1980-220X-REEUSP-2022-0330en

**Published:** 2023-01-20

**Authors:** Lucas Verzegnassi Vieira, Vinicius Lino de Souza, Alba Lúcia Bottura Leite de Barros, Juliana de Lima Lopes, Leticia Fernanda Tavares Sousa de Oliveira, Mariana Alvina dos Santos, Camila Takao Lopes, Vinicius Batista Santos

**Affiliations:** 1 Universidade Federal de São Paulo, Escola Paulista de Enfermagem, São Paulo, SP, Brazil.; 2 Universidade Federal de Mato Grosso do Sul. Campus Três Lagoas, Três Lagoas, MS, Brazil.

**Keywords:** Coronary Diseas, Health Educatio, Validation Stud, Text Messaging, Doença das Coronárias, Educação em Saúde, Estudo de Validação, Envio de Mensagens de Texto, Enfermedad Coronaria, Educación en Salud, Estudio de Validación, Envío de Mensajes de Texto

## Abstract

**Objective:**

To identify information needs of patients with coronary artery disease and develop and validate the content of educational messages for mobile phones for these patients.

**Method:**

The study was carried out in three phases: 1) Identification of information needs in relation to coronary artery disease of patients hospitalized for an acute coronary event; 2) Development of templates containing text and pictures about the disease and treatment; 3) Content validity analysis of template evidence through the assessment of 10 experts. Templates were considered validated when the Content Validity Ratio (CVR) was equal to or greater than 0.80.

**Results:**

A total of 67 patients were included, and all the information that emerged about the disease was classified as important to very important. Thirty templates were developed (heart function, recommendations on nutrition and exercise, treatments and medications, and clinical signs related to the disease and risk factor control), and the CVR obtained was greater than 0.80.

**Conclusion:**

All information needs were categorized by patients as important or very important. The templates were developed and validated considering content and design.

## RESUMO

**Objetivo:**

Identificar as informações sobre necessidades de pacientes com doença arterial coronariana e desenvolver e validar o conteúdo de mensagens educativas para celulares para esses pacientes.

**Método:**

O estudo foi realizado em três fases: 1) Identificação das informações sobre necessidades em relação à doença arterial coronariana de pacientes internados por evento coronariano agudo; 2) Desenvolvimento de templates contendo textos e imagens sobre a doença e tratamento; 3) Análise da validade de conteúdo das evidências do template por meio da avaliação de 10 especialistas. Os templates foram considerados validados quando a Razão de Validade de Conteúdo (RVC) foi igual ou superior a 0,80.

**Resultados:**

Foram incluídos 67 pacientes, e todas as informações que surgiram sobre a doença foram classificadas como importantes a muito importantes. Foram desenvolvidos 30 templates (função cardíaca, recomendações de nutrição e exercícios, tratamentos e medicamentos, sinais clínicos relacionados à doença e controle de fatores de risco), e a RVC obtida foi superior a 0,80.

**Conclusão:**

Todas as necessidades de informação foram categorizadas pelos pacientes como importantes ou muito importantes. Os templates foram desenvolvidos e validados considerando conteúdo e design.

## RESUMEN

**Objetivo:**

Identificar las necesidades de información de los pacientes con enfermedad de las arterias coronarias y desarrollar y validar el contenido de mensajes educativos para teléfonos celulares para estos pacientes.

**Método:**

El estudio se realizó en tres fases: 1) Identificación de necesidades de información sobre enfermedad de las arterias coronarias en pacientes hospitalizados por evento coronario agudo; 2) Desarrollo de templates que contienen textos e imágenes sobre la enfermedad y el tratamiento; 3) Análisis de la validez de contenido de la evidencia del template a través de la evaluación de 10 expertos. Los templates se consideraron validadas cuando el Razón de Validez de Contenido (RVC) fue igual o superior a 0,80.

**Resultados:**

Se incluyeron 67 pacientes y toda la información que surgió sobre la enfermedad se clasificó como importante a muy importante. Se desarrollaron 30 templates (función cardiaca, recomendaciones de nutrición y ejercicio, tratamientos y medicamentos, signos clínicos relacionados con la enfermedad y control de factores de riesgo), y la RVC obtenida fue superior a 0,80.

**Conclusión:**

Todas las necesidades de información fueron categorizadas por los pacientes como importantes o muy importantes. Los templates fueron desarrollados y validados considerando contenido y diseño.

## INTRODUCTION

Cardiovascular diseases are the main cause of morbidity, disability and death in the world^(1)^. Among cardiovascular diseases, coronary artery disease (CAD) stands out. This is a chronic disease that has resulted in a constant increase in hospital admission rates for acute events, such as acute myocardial infarction (AMI) or other ischemic heart diseases^(1,2)^. In this context, it is necessary that individuals with CAD carry out self-management of pharmacological and non-pharmacological treatment in order to ensure the reduction of atherosclerotic progression, preventing new coronary events, stenosis or thrombosis of coronary stents or vascular grafts^(2,3,4,5)^.

To support patient self-management, health professionals can implement various educational interventions aimed at controlling cardiovascular risk factors, correct use of medications and incorporation of a healthy lifestyle^(3–6)^ through periodic telephone consultations, face-to-face multidisciplinary consultations, use of educational messages for mobile phones, educational games and applications for mobile devices^(3–6)^. For individuals with CAD, the use of short messages for smartphones has been associated with an improvement in LDLc levels, blood pressure levels, Body Mass Index (BMI), physical activity levels, smoking, knowledge levels, depressive symptoms and compliance with pharmacological treatment^(7,8,9,10,11)^.

As much as we already have national and international guidelines on secondary prevention of CAD, the development of educational messages demands awareness of information needs on the disease and its prevention, considering the educational and cultural differences in Brazil and in the world. However, in Brazil, to the best of our knowledge, there are no studies that assessed the need for information on secondary prevention for individuals with CAD, considering the regional differences around the world. This study aimed to identify information needs of patients with CAD, in addition to developing and validating the content of educational messages for mobile phones for these patients.

## METHODS

### Study Design

This study was conducted in three phases: 1) Assessment of information needs of patients with CAD regarding secondary prevention; 2) Elaboration of educational text messages; 3) Message content validity. The first phase consisted of an observational, analytical and cross-sectional study. The second and third phases consisted of a methodological study of content development and educational material validity.

PHASE 1: ASSESSMENT OF INFORMATION NEEDS OF PATIENTS WITH CORONARY ARTERY DISEASE REGARDING SECONDARY PREVENTION

### Study Location

Data collection was performed in cardiac units (Cardiac Unit, Cardiac Intensive Care Unit) and in the hemodynamic service of a large public university hospital in the city of São Paulo, SP, Brazil, from December 2019 to March 2020.

### Population and Sample

The study population consisted of all patients hospitalized for any manifestation of CAD (acute coronary syndrome or stable angina) in the institutions. Patients over 18 years of age, without a previous diagnosis of dementia or neuropsychiatric diseases and without clinical signs of severe acute ventricular dysfunction (acute pulmonary edema or cardiogenic shock) or who, at the time of data collection, presented and/or ejection fraction at hospitalization less than or equal to 40%, were included.

### Variables of Interest and Data Collection

Potentially eligible patients were approached and received an explanation about the research objectives. Upon acceptance and signing of the Informed Consent Form, sociodemographic (gender, age, religion, education, monthly income and race) and clinical characteristics (medical admission diagnosis and comorbidities) were extracted from the medical records.

The CAD information needs assessment considered the Information Needs in Cardiac Rehabilitation (INCR)^(12)^ instrument, validated for Brazilian Portuguese^(13)^. The Brazilian version of INCR contains 55 questions, arranged in ten subscales representing information needs: The heart (physiology, symptoms, surgical treatments), Nutrition, Exercise/physical activity, Medication, Work/vocational/social, Stress/psychological factors, General/social concerns, Emergency/safety, Diagnosis and treatment, Risk factors. For each question, patients assessed the level of importance in having the information, ranging from 1 (completely not important), 2 (not important), 3 (neutral), 4 (important) and 5 (very important)^(12,13)^. The total score ranges from 55 points to 275 points. The higher the score, the greater the need for information. To understand the specific need for information, each item should be individually assessed.

The original instrument was submitted to psychometric validity^(12)^ with 20 experts, 20 patients in pre-test and 203 participants, demonstrating an internal consistency assessed by Cronbach’s alpha of 0.80 for each area of information needs. In the study of psychometric validity in Portuguese^(13)^, an internal consistency assessed by Cronbach’s alpha of 0.71 to 0.91 was also obtained, depending on the subscale assessed.

PHASE 2: ELABORATION OF EDUCATIONAL TEXT MESSAGES

After identifying information needs, text and image messages were developed addressing the issues considered most important in the previous phase. The messages were developed based on the main international guidelines^(2,3,6)^ on secondary prevention for patients with CAD by a nursing student, under the supervision of a nurse who specializes in cardiology, PhD, with 20 years of practical experience, teaching, and research experience.

These messages were constructed using informal language, with short sentences, and illustrations were developed by a professional illustrator to improve the understanding of the elaborated sentences. To develop the messages, we used a software, Canva^®^, with Arial 23-point font, 1.5 line spacing, white background, borders simulating a whiteboard. In all messages there were two fictitious characters named Nurse Julia and Cardiolino, an anthropomorphized heart with the logo of the extension project in which this study was developed.

The messages developed were divided into 8 domains that were based on the instrument used in phase 1^(12,13)^, but Risk factors, Psychosocial factors and General/social concerns domains were gathered in only one domain.

PHASE 3 ANALYSIS OF CONTENT VALIDITY EVIDENCE OF EDUCATIONAL TEXT MESSAGES

Ten professionals were invited to assess the messages developed: seven nurses and one nutritionist with experience of at least 2 years in cardiology, and two linguists (Portuguese teachers). Data were collected from April to December 2020.

Experts were selected based on the researchers’ prior knowledge. An invitation to participate in the study was sent by email. Upon signing the Informed Consent Form, a second email was sent with the link to fill in the data collection instruments through Google Forms^®^.

Experts were asked to assess the messages for clarity of sentences, practical relevance, font size and type, images distinctness and relationship of images to the text, using a four-point Likert-type scale: 1 = totally inadequate, 2 = partially inadequate, 3 = partially adequate, and 4 = completely adequate.

### Ethical Aspects

The project was submitted and approved by the local Research Ethics Committee on Human Beings, under Opinion 3.343.733, in 2019. The study met all scientific requirements according Resolution 466/2012 and the Declaration of Helsinki.

### Data Analysis

Descriptive statistics were used to characterize the sample of patients and experts. Categorical measures were expressed as absolute (n) and relative (%), and frequencies and continuous variables, as means and standard deviation (SD).

The Content Validity Ratio (CVR) was calculated in relation to experts’ opinions through the agreement of items. The CVR was calculated as follows: *CVR: ne- (N/2)/ N/2* (*CVR* is the Content Validity Ratio; *ne* is the number of panel members indicating an item “adequate”; and *N* is the number of panel members). Considering the sample of 10 experts, for the critical CVR to be considered ideal, the values must be above 0.80 considering a significance level of 0.011^(14)^.

## RESULTS

In total, 67 patients were included in phase 1 of the study, whose sociodemographic and clinical characteristics are shown in Table 1.

**Table 1.** Sociodemographic and clinical characteristics of patients with coronary artery disease, n = 67 – São Paulo, SP, Brazil, 2020.

Variable	Total
***Gender (male), n(%)***	47 (70.1)
***Age, mean (SD)***	62.39 (10.22)
***Color, n(%)***	
White	43 (64.2)
Yellow	04 (6.0)
Black	20 (29.9)
***Religion, n(%)***	
Catholicism	35 (52.2)
Evangelical	17 (25.4)
Spiritist	02 (3.0)
None	12 (19.4)
***Schooling% (n)***	
Incomplete Elementary School	27 (40.3)
Complete Elementary School	06 (9.0)
Incomplete High School	06 (9.0)
Complete High School	19 (28.4)
Incomplete Higher Education	01 (1.5)
Complete Higher Education	08 (11.9)
***Family income (minimum wages), n(%)***	
Up to 3	36 (53.7)
3 to 5	12 (17.9)
5 to 7	15 (22.4)
More than 9	04 (6.0)
***Marital Status***	
Divorced	10 (14.9)
Married	42 (62.7)
Single	06 (9.0)
Widow	02 (3.0)
Common-law marriage	07 (10.4)
***Medical diagnoses***	
Stable angina	16 (23.9)
Unstable angina	02 (3.0)
AMI without ST elevation	29 (43.2)
ST-segment elevation myocardial infarction	20 (29.9)
***Comorbidities – n (%)***	
Hypertension	44 (65.7)
Diabetes Mellitus	23 (34.3)
Dyslipidemia	17 (25.4)
Stroke	03 (4.5)
Previous AMI	11 (16.4)
***Risk factors***	
Smoking	17 (25.4)
Alcohol consumption	14 (20.9)
Sedentary lifestyle	42 (62.7)
Body Mass Index, mean (SD)	25.23 (9.0)

SD: standard deviation.

Table 2 shows that all INCR domains were considered important to very important, especially The heart, Emergency/safety, General/social concerns and Diagnosis and treatment domains. The most important information needs were: “What medications do I need to help my heart?”; “When should I call the doctor?”; “What happens when someone has a heart attack (infarction)?”; “How does a healthy heart work?”; “What should I do if I feel angina or chest pain?”; and “How can cholesterol and diabetes mellitus affect my heart?” (Table 2).

**Table 2.** Assessment of information needs of patients with coronary artery disease by Information Needs in Cardiac Rehabilitation (INCR) – São Paulo, SP, Brazil, 2020.

Question	Mean (SD)
**Subscale 1 (The heart)**	4.69 (0.52)
1. How does a healthy heart works?	4.79 (0.68)
2. What is “coronary artery disease?”	4.56 (0.97)
3. What is angina?	4.59 (0.97)
4. What happens when someone has a heart attack (infarction)?	4.80 (0.60)
5. What is “bypass surgery” (bridge saphenous vein)?	4.70 (0.71)
6. What is an angioplasty (stent)?	4.70 (0.83)
**Subscale 2 (Nutrition)**	4.57 (0.60)
7. What foods should I eat for a healthy heart?	4.68 (0.85)
8. How can I choose healthy foods at the grocery store?	4.77 (0.54)
9. How can I choose healthy foods when dining out?	4.49 (1.07)
10. How do I read food labels?	4.35 (1.02)
**Subscale 3 (Exercise/physical activity)**	4.38 (0.80)
11. How will exercise help my heart condition?	4.71 (0.79)
12. What are the components of a safe exercise program?	4.56 (0.95)
13. What is cardiovascular or aerobic exercise?	4.44 (1.06)
14. What can I do to improve or maintain flexibility?	4.50 (0.85)
15. How should I exercise in hot or cold weather?	4.34 (1.13)
16. If I have diabetes, how do I prevent low blood sugar with exercise?	4.50 (0.89)
17. How do I take care for my feet when in an exercise program?	4.37 (1.02)
18. What is resistance training (i.e., exercise to strengthen)?	3.97 (1.46)
19. What types of exercise equipment are available? (where?)	3.97 (1.45)
20. How can I exercise at home safety?	4.46 (1.01)
21. When should I stop physical exercise?	4.47 (1.10)
22. Is sexual activity safe for me?	4.35 (1.13)
**Subscale 4 (Medication)**	4.53 (0.73)
23. What medications do I need to help my heart?	4.83 (0.70)
24. How do I take my medication in the right way?	4.70 (0.79)
25. What side effects are possible with my medication?	4.61 (0.86)
26. Do the medications I am taking interfere with each other?	4.52 (0.97)
27. Are there foods I should avoid while taking these medications?	4.38 (1.08)
28. What are the effects of complementary and alternative medications?	4.17 (1.19)
Subscale 5 (Work/vocation/social)	4.24 (1.01)
29. When can I go back to work and my old activities?	4.55 (1.04)
30. Can I go back to the same job?	4.35 (1.16)
31. When can I start driving again?	3.83 (1.65)
**Subscale 6 (Stress/psychological factors)**	4.55 (0.79)
32. What feelings are common after a heart attack (infarction)?	4.53 (0.95)
33. How does stress affect my heart?	4.71 (0.73)
34. How can I cope with stress?	4.55 (0.95)
35. What can I do to reduce stress in my life?	4.50 (1.0)
36. Do sleep problems affect my heart?	4.47 (0.99)
**Subscale 7 (General/social concerns)**	4.66 (0.73)
37. What services, support organizations and groups are available?	4.61 (0.92)
38. What support services are available to my family?	4.71 (0.81)
**Subscale 8 (Emergency/safety)**	4.67 (0.68)
39. How do I recognize angina symptoms?	4.47 (1.03)
40. What should I do if I feel angina or chest pain?	4.73 (0.75)
41. When should I call the doctor?	4.82 (0.62)
42. When should I call 192 (SAMU) or go to emergency room?	4.67 (0.74)
**Subscale 9 (Diagnosis and treatment)**	4.64 (0.72)
43. What are the tests used to diagnosis my heart condition?	4.58 (0.72)
44. What treatments are available for my condition?	4.71 (0.76)
**Subscale 10 (Risk factors)**	4.62 (0.76)
45. What are the risk factors for heart disease?	4.65 (0.80)
46. What are the risk factors that I cannot control?	4.62 (0.88)
47. What are the risk factors I can control?	4.58 (1.01)
48. What can I do to bring my risk factors under control?	4.64 (0.94)
49. How does cholesterol affect my heart?	4.73 (0.75)
50. How does diabetes affect my heart?	4.73(0.77)
51. How does physical inactivity affect my heart?	4.68 (0.74)
52. How does smoking affect my heart?	4.61 (1.02)
53. What are the benefits of quitting smoking?	4.55 (1.14)
54. What supports are available to help me quit smoking?	4.47 (1.13)
55. How does alcohol affect my heart?	4.58 (1.04)
**Total**	**4.56 (0.62)**

SD: Standard Deviation.

**Figure 1.**
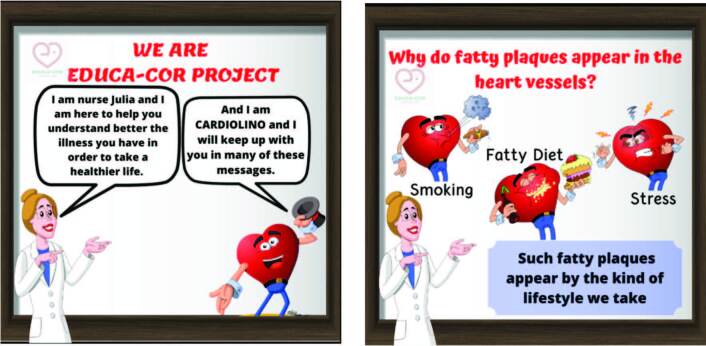
Examples of developed and validated templates.

Based on patients’ information needs, 30 templates were developed, divided into 8 domains, containing text messages and images to guide and motivate patients with CAD (Supplementary File 1). The messages were built with two fictional characters: the nurse Julia and the caricature Cardiolino (an anthropomorphized heart). Figure 1 shows the two templates containing the presentation of the characters.

In the domain related to knowledge of the heart and its functioning, two messages were constructed that aim to guide patients in relation to the heart’s physiological process and CAD repercussions for the heart muscle. In the Nutrition domain, two messages were developed with guidance on the most suitable foods for patients with CAD and the method of preparation. In this domain, there were suggestions by experts for changes in relation to the images, as some of them contained foods not suitable for this population.

**Figure 2.**
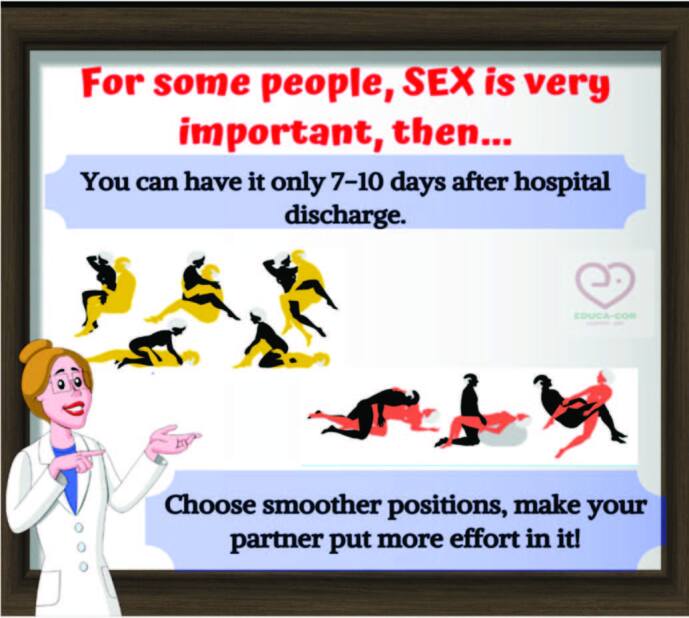
Template related to sexual activities.

Regarding the Physical activity domain, six messages were developed. These messages provide guidelines regarding safe practice in performing the exercises, such as the clothing to be used, types of exercises, indications for stretching, minimum time to perform exercises and what symptoms patients should notice when performing physical exercises. This domain also included a message related to sexual activities to guide patients on the safe return to sexual practice (Figure 2).

For the Medication domain, four messages were developed, guiding patients on the main medications they are normally prescribed for their possible side effects, medication use duration and the main ways to remember to take them. In the Work domain, a message was constructed that addresses the time indicated for returning to work activities and the importance of discussion with a health team regarding this return. In the Emergency domain, two messages were constructed focusing on the possible symptoms of myocardial ischemia that patients must be aware of, what symptoms may appear during physical activities and procedures to be followed.

For the Diagnosis domain, four messages were elaborated, which provide guidance on therapeutic possibilities for patients with CAD, detailing clinical treatment, percutaneous coronary intervention and surgical myocardial revascularization. In the Risk factors and General/social concerns domains, eight templates were built related to changes in lifestyle, mainly in relation to tobacco control and alcohol consumption. The developed templates were sent and answered by 10 experts. Table 3 shows the mean of CVR values for each domain. All CVRs for each domain were presented with values equal to or greater than those recommended in relation to the number of experts.

**Table 3.** Content Validity Ratio of each domain and the global score in relation to assessed indicators – São Paulo, SP, Brazil, 2020.

Domains	Clarity	Pertinence	Font size and type	Image distinctness	Relation of images to the text
The heart	1.0	1.0	1.0	1.0	1.0
Nutrition	0,80	1.0	1.0	1.0	0.80
Physical activity	1.0	0.80	1.0	1.0	1.0
Medication	0,80	0,80	0,80	0,80	0,80
Work	1.0	1.0	1.0	1.0	1.0
Emergency/safety	1.0	1.0	1.0	1.0	1.0
Diagnosis and treatment	1.0	1.0	1.0	1.0	1.0
Risk factors and General/social concerns	1.0	0.9	1.0	1.0	0.9

## DISCUSSION

Knowing patients’ information needs is the first step in health education^(15,16)^. This educational process is characterized by a systematic, sequential, logical, planned and scientifically based course of action. It consists of two main interdependent operations, teaching and learning, which, together play their roles, the result of which leads to changes in mutually desired behaviors. This educational process aims to achieve changes in individuals’ attitudes and skill, something that is only possible with adequate instrumentalization of patients and families^(15)^.

Given the need to identify patients’ main doubts, this study was initially conducted to know the main information needs of coronary patients to guide the development of text and image messages.

To analyze information needs of patients in relation to CAD, the instrument called “Information Needs in Cardiac Rehabilitation” was used. It was developed in English^(12)^ and validated in Portuguese^(13)^, Spanish^(17)^ and Chinese^(18)^. The construction of this instrument^(12)^ was carried out after reviewing the literature and submitting it to content validity with 10 experts. Its psychometric properties were assessed with 203 patients, obtaining a Cronbach’s alpha value above 0.70 and valid for Brazil. This study was submitted to 300 patients and adequate validity evidence was obtained regarding internal consistency.

The mean scores of importance attributed to information about cardiac rehabilitation in our study were higher when compared to the means obtained in the original study^(12)^ (4.58 vs 4.08) and in the validity study for Brazil^(13)^ (4.58 vs 4.08), but in all these studies patients considered indicators as important to very important.

In the study on the Chinese questionnaire translation^(18)^, with a population of 296 participants, similar findings to ours were identified, except in the Stress/psychological factors and General/social concerns domains, in which the means of the importance attributed to these domains were 4.55 and 4.66 (important to very important), respectively. In our study and in the Chinese one, it was less than 4 points, considering it does not matter to important^(18)^. These comparisons reinforce the relevance of assessing the need for information in each context where professionals are inserted, because a population’s needs may not be the same as those of other individuals.

The domains with the greatest need for information were General/social concerns and Emergency/safety, which can be explained by patients’ fear of their health, lack of information about actions to be taken in view of a new CAD episode, and low socioeconomic level, given the fact they usually need information about support groups and associations.

The messages developed in response to information needs involved issues related to secondary prevention in coronary heart disease, i.e., understanding of the disease, management of precordial pain recurrence, nutritional and physical activity recommendations, including sexual activity, measures to improve medication compliance, guidelines for work activities and control of habits that impact the progression of atherosclerotic diseases such as smoking, alcohol consumption and stress.

Many of these messages, particularly those related to the adequacy or incorporation of a healthy lifestyle, are also supported by the American Heart Association’s health checklist recommendations, which include 7 essential health behaviors for the general population (smoking, physical activity, diet and weight) and health factors (cholesterol control, blood pressure and glucose control) that contribute to cardiovascular health^(19)^.

The development of text messages for mobile phones is one of the great advances in technology for use in secondary prevention programs in CAD^(20–24)^, because direct supervision programs currently have low patient compliance due to the lack of interest in group activities, geographic distances to the program location, lack of parking at program locations and need to return to work activities^(25)^.

Studies with coronary patients demonstrate that using text messages to mobile phones can increase pharmacological and non-pharmacological compliance levels^(22–24)^. The TEXT ME study included 710 patients with CAD who received messages related to smoking, exercise and diet for 6 months. The study showed that patients who received the messages had lower LDLc levels, lower blood pressure, lower BMI, higher physical activity, lower smoking rate and fewer symptoms of mild- to-moderate depression^(23)^.

Significant data were also identified in the CHAT-DM study, with 502 coronary patients with DM, who received 6 messages per week for 6 months. Patients had lower glycated hemoglobin levels (p. 0.003) and there was a higher proportion of patients who achieved the recommended levels of HbA1c^(21)^. In the Text4Heart study, daily SMS text messages and a supporting website significantly supported compliance with healthy lifestyle behaviors of patients with coronary heart disease for up to 3 months, as measured by a self-reported composite health behavior score^(26)^.

The messages developed were submitted to content validity evidence analysis by a committee of experts, in order to be reliably used in the population of coronary patients. In a recent literature review, it was shown that using valid and reliable instruments can lead to better diagnostic accuracy and implementation of preventive strategies^(27)^. The main suggestions made by experts, which were accepted by the researchers, were related to the suggestion of the most common and cheapest types of food for the Brazilian population; changes in the writing and standardization of terms (e.g., CAD); and suggestion of some types of physical exercises to avoid doubts in reading the information.

The committee developed in this study followed the recommendations regarding expert quantity and quality, based on clinical experience in the care of coronary patients and on language expertise to improve the clarity of information^(28)^. The messages in our study were based on the responses of a previously validated instrument, but data were collected in a single center, where patients in general have low income. Therefore, a limitation of our study is that we cannot infer that our messages will meet generalized needs of patients assisted in other centers in the Brazilian territory or even in the world population. Follow-up studies will be conducted to validate cognitive testing with patients with CAD and assess the effectiveness of using these messages in lifestyle modification and medication compliance.

## CONCLUSIONS

Information needs related to CAD were assessed as important to very important by patients in all analyzed domains, mainly related to The heart and Emergency/safety.

In total, 30 templates were developed, covering information on the main issues related to secondary prevention of CAD. Adequate content validity evidence was obtained according to expert opinion.

## References

[1] Virani SS, Alonso A, Benjamin EJ, Bittencourt MS, Callaway CW, Carson AP (2020). Heart disease and stroke statistics-2020 update: a report from the American Heart Association. Circulation..

[2] Urbinati S, Tonet E (2018). Cardiac rehabilitation after STEMI. Minerva Cardioangiol..

[3] Piepoli MF, Corrà U, Dendale P, Frederix I, Prescott E, Schmid JP (2017). Challenges in secondary prevention after acute myocardial infarction: a call for action. Eur J Cardiovasc Nurs..

[4] Kim HY, Kim SH, Jung HJ, Kim HS (2019). Development and evaluation of self-management program for patients with coronary artery disease. J Multimed Inf Syst..

[5] Farias MS, Silva LF, Brandão MAG, Guedes MVC, Pontes KMA, Lopes ROP (2021). Medium reach theory for nursing in cardiovascular rehabilitation. Rev Bras Enferm..

[6] Visseren FLJ, Mach F, Smulders YM, Carballo D, Koskinas KC, Bäck M (2021). 2021 ESC Guidelines on cardiovascular disease prevention in clinical practice. Eur Heart J..

[7] Schwaab B (2018). Cardiac rehabilitation. Rehabilitation (Stuttg)..

[8] Hamilton SJ, Mills B, Birch EM, Thompson SC (2018). Smartphones in the secondary prevention of cardiovascular disease: a systematic review. BMC Cardiovasc Disord..

[9] Shariful Islam SM, Farmer AJ, Bobrow K, Maddison R, Whittaker R, Pfaeffli Dale LA (2019). Mobile phone text-messaging interventions aimed to prevent cardiovascular diseases (Text2PreventCVD): systematic review and individual patient data meta-analysis. Open Heart..

[10] Indraratna P, Tardo D, Yu J, Delbaere K, Brodie M, Lovell N (2020). Mobile phone technologies in the management of ischemic heart disease, heart failure, and hypertension: systematic review review and meta-analysis. JMIR Mhealth Uhealth..

[11] Shariful Islam SM, Chow CK, Redfern J, Kok C, Rådholm K, Stepien S (2019). Effect of text messaging on depression in patients with coronary heart disease: a substudy analysis from the TEXT ME randomised controlled trial. BMJ Open..

[12] Ghisi GLM, Grace SL, Thomas S, Evans MF, Oh P (2013). Development and psychometric validation of a scale to assess information needs in cardiac rehabilitation: the INCR Tool. Patient Educ Couns..

[13] Ghisi GLM, Santos RZ, Bonin CBD, Roussenq S, Grace SL, Oh P (2014). Validation of a Portuguese version of the Information Needs in Cardiac Rehabilitation (INCR) scale in Brazil. Heart Lung J Acute Crit Care..

[14] Ayre C, Scally AJ (2014). Critical values for lawshe’s content validity ratio: revisiting the original methods of calculation. Meas Eval Couns Dev..

[15] Murdaugh CL, Parsons MA, Pender NJ (2018). Health promotion in nursing practice..

[16] Ferreira PBP, Porto IS, Santo FHDE, Figueiredo NMA, Enders BC, Cameron LE (2021). Health education for hospitalized patient in nursing care: a conceptual analysis. Rev Bras Enferm..

[17] Ghisi GLM, Anchique CV, Fernandez R, Quesada-Chaves D, Gordillo MX, Acosta S (2018). Validation of a spanish version of the information needs in cardiac rehabilitation scale to assess information needs and preferences in cardiac rehabilitation. J Cardiovasc Nurs..

[18] Ma C, Yang Q, Huang S (2019). Translation and psychometric evaluation of the chinese version of the information needs in cardiac rehabilitation tool. J Cardiopulm Rehabil Prev..

[19] American Heart Association (2022). My Life Check – Life’s Simple 7 [Internet].

[20] Taylor RS, Dalal HM, McDonagh STJ (2022). The role of cardiac rehabilitation in improving cardiovascular outcomes. Nat Rev Cardiol..

[21] Huo X, Krumholz HM, Bai X, Spatz ES, Ding Q, Horak P (2019). Effects of mobile text messaging on glycemic control in patients with coronary heart disease and Diabetes Mellitus: a randomized clinical trial. Circ Cardiovasc Qual Outcomes..

[22] Chen S, Gong E, Kazi DS, Gates AB, Bai R, Fu H (2018). Using mobile health intervention to improve secondary prevention of coronary heart diseases in China: mixed-methods feasibility study. JMIR Mhealth Uhealth..

[23] Shariful Islam SM, Chow CK, Redfern J, Kok C, Rådholm K, Stepien S (2019). Effect of text messaging on depression in patients with coronary heart disease: a substudy analysis from the TEXT ME randomised controlled trial. BMJ Open..

[24] Adler AJ, Martin N, Mariani J, Tajer CD, Owolabi OO, Free C (2017). Mobile phone text messaging to improve medication adherence in secondary prevention of cardiovascular disease. Cochrane Database Syst Rev..

[25] Thomas RJ, Beatty AL, Beckie TM, Brewer LC, Brown TM, Forman DE (2019). Home-based cardiac rehabilitation: a scientific statement from the american association of cardiovascular and pulmonary rehabilitation, the American Heart Association, and the American College of Cardiology. J Am Coll Cardiol..

[26] Pfaeffli Dale L, Whittaker R, Jiang Y, Stewart R, Rolleston A, Maddison R (2015). Text message and internet support for coronary heart disease self-management: results from the Text4Heart randomized controlled trial. J Med Internet Res..

[27] Hanskamp-Sebregts M, Zegers M, Vincent C, van Gurp PJ, de Vet HC, Wollersheim H (2016). Measurement of patient safety: a systematic review of the reliability and validity of adverse event detection with record review. BMJ Open..

[28] Almanasreh E, Moles R, Chen TF (2019). Evaluation of methods used for estimating content validity. Res Social Adm Pharm..

